# Live-cell physiology in human brain tissue culture—the potential, the challenges, and the lessons learned

**DOI:** 10.3389/fncel.2026.1777650

**Published:** 2026-04-30

**Authors:** Marc Oudart, Karen M. J. van Loo, Deborah Kronenberg-Versteeg

**Affiliations:** 1 German Center for Neurodegenerative Diseases (DZNE), Tübingen, Germany; 2 Department of Cellular Neurology, Hertie Institute for Clinical Brain Research, University of Tübingen, Tübingen, Germany; 3 Department of Epileptology, Neurology, RWTH Aachen University Hospital, Aachen, Germany; 4 Department of Neurosurgery, RWTH Aachen University Hospital, Aachen, Germany

**Keywords:** brain slice culture, cell physiology, disease modeling, human brain tissue, virus labeling

## Abstract

A growing number of studies are now utilizing human organotypic brain slice cultures (OBSC), enabled by improved long-term culture protocols that support the investigation of human-specific cellular physiology. Human OBSC offer the unique advantage, that they preserve the connectivity and microenvironment of human brain tissue while remaining experimentally tractable for live functional assays. However, methods established in rodent brain slice cultures, such as viral labeling, genetic manipulation, and long-term transduction strategies, do not readily translate to human tissue due to differences in viral tropism, tissue heterogeneity, and limited survival in culture. In this review, we highlight key historical developments and provide an overview of current best practices of human brain tissue culture. We provide an overview of live cell-specific labeling strategies, including viral approaches, as well as current and emerging technologies for functional manipulation and for investigating disease-related pathologies. Throughout, we integrate insights gained from mouse models and discuss how these technologies could potentially be adapted and leveraged for human OBSCs. Beyond methodological approaches, we also address the challenges and limitations inherent to working with human brain tissue. Together, these advances position human OBSCs as a powerful platform to bridge the gap between reductionist *in vitro* systems and the *in vivo* living human brain.

## 1 History of human organotypic brain slice cultures

**Figure 1:**
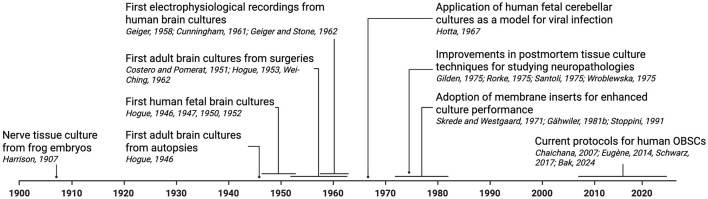
Historical timeline of human organotypic brain slice culture development, including cross-species milestones. Created with BioRender.com.

Human organotypic brain slice cultures (OBSCs) have emerged as a powerful *ex vivo* model bridging acute slice physiology, rodent models, and human brain organoids. They preserve local cytoarchitecture and connectivity, making them invaluable for studying human-specific neurobiology and disease mechanisms. The exploration of brain slice culture has a long history (Figure 1), dating back to the early twentieth century. Ross G. Harrison first cultured nerve tissue from frog embryos in 1907 to study neurite outgrowth

(Harrison, 1907), laying groundwork for later developments in tissue culture. In the mid-twentieth century, Mary Jane Hogue pioneered human fetal brain culture (for a detailed biography and review of her work, see Zottoli and Seyfarth, 2015, 2018; Figure 1), using hanging-drop culturing techniques as adapted from Lewis and Lewis' embryology work (Lewis and Lewis, 1915). This allowed Hogue to study the migration of nervous cells effectively, utilizing specimens from early developmental stages, including fetal, infant, and adult human brains as described in the following paragraphs. In her early paper from 1946, Hogue used brightfield and darkfield microscopy along with histological stainings (e.g., neutral red and Sudan III) to study “granules” (which likely were polyribosomes), and described neuronal and glial motility in various animal and human brain tissue (Hogue, 1946). She also reported human fetal nerve cells surviving in culture for up to 143 days (this experiment was unfortunately prematurely terminated due to a malfunctioning incubator; Hogue, 1947). Hogue later adopted roller-tube cultures to improve handling and nutrient flow, and documented viability and migration of cells in both fetal and adult human surgical tissue (Hogue, 1950, 1952, 1953).

The early 1950s brought further advancement when Hogue (1953) successfully utilized adult surgical tissues, documenting living brain cells that had migrated from the slice into the culture dish after 60 days (Figure 1). Her investigations also included studying choroid plexus cells and the impact of viral infection on brain cultures (Hogue, 1949; Hogue et al., 1955). At the same time as Hogue was pioneering, Pomerat (1946) was conducting his own research, focusing on mammalian brain tissue cultures (Lumsden and Pomerat, 1951; Pomerat and Costero, 1956). Intriguingly, they also reported roller-tube derived neuronal cultures from migrating cells of adult human tissue cultures obtained from 8,000 explants of 121 patients (Costero and Pomerat, 1951). In addition, Geiger (1958), Cunningham (1961), and Geiger and Stone (1962) expanded mammalian slice culture techniques and began to demonstrate neuronal growth and neural activity in culture, including the first electrophysiological recordings from human cultures. Around the same time, Wei-ching (1962) managed to culture adult human brain tissue from neurosurgical operations for an impressive 5 months, and Hotta et al. (1967) explored human fetal cerebellar cultures, diving into the implications of viruses like Japanese encephalitis.

From the 1970s onwards, improved media formulations, flask cultures, and the establishment of immortalized cell lines using viral vectors, advanced the field and enabled studies of culturing postmortem adult human tissue to investigate neuropathologies such as multiple sclerosis, Creutzfeldt-Jakob disease, and amyotrophic lateral sclerosis (ALS; Gilden et al., 1975; Rorke et al., 1975; Santoli et al., 1975; Wroblewska et al., 1975). In the 1980s−1990s the organotypic slice technique matured: thinner slices (300–500 μm) improved diffusion and viability (Skrede and Westgaard, 1971; Gähwiler, 1981b) and the use of air-medium interface methods using membrane inserts (Stoppini et al., 1991) enhanced oxygenation and long-term maintenance. Using these adaptations, Gähwiler was the first to label neurons in living rat brain slice cultures using intracellular dyes (lucifer yellow; Gähwiler, 1981a). By the mid-1990s, viral strategies for infecting brain tissue had advanced substantially, including early work using Vaccinia virus to transfect neurons in rat hippocampal slice cultures (Pettit et al., 1995) and adenoviral delivery of the *Lac*Z gene *in vivo* (Akli et al., 1993), which only a few years later was successfully applied to human brain slice cultures (O'Connor et al., 1997). While these early studies demonstrated the feasibility of culturing human brain tissue *ex vivo*, further advances in slice culture preparations were necessary to enable robust and functional experimentation.

## 2 Methods and innovations for human OBSC

### 2.1 Current methods of cultivating human brain slices

Establishing reliable protocols for human OBSCs has been crucial for expanding their potential as an experimental platform. Compared to rodent OBSCs, which can survive for several months and are highly amenable to genetic manipulation (Humpel, 2015; Croft and Noble, 2018; Croft et al., 2019), human brain slices face more stringent constraints due to variable tissue quality, limited access, and reduced long-term maintenance (See Challenges and Limitation section). Culture parameters, ranging from tissue source (postmortem vs. surgical resection) and patient pathology to slice thickness, medium composition, and whether slices are maintained at an air-medium interface or in free-floating systems, critically determine viability, cytoarchitecture, and experimental reproducibility.

Early work by Chaichana et al. (2007) showed that adult human OBSCs could be maintained for up to 14 days using ≤ 350 μm slices and a glucose-enriched MEM-based medium with daily replacement (Chaichana et al., 2007). Building on this foundation, Richard Miles' lab introduced a medium that mimics human cerebrospinal fluid (CSF), allowing hippocampal OBSCs from epileptic patients to be cultured for up to 3–4 weeks without altering ictal-like activity (Eugène et al., 2014). Schwarz et al. (2017) further demonstrated that human CSF enhances cortical slice health and electrophysiological stability compared to classical media such as MEM or BME (Schwarz et al., 2019).

Other refinements have expanded the methodological repertoire. Fernandes et al. (2019) provided a short-term, cost-effective free-floating protocol allowing survival up to 4 days without membranes (Fernandes et al., 2019), whereas Kvist (2021) used BrainPhys medium to maintain cortical slices from epilepsy patients for up to 6 weeks based on FOX3 and SC121 markers and electrophysiological data (Kvist, 2021). Most recently, Bak et al. (2024) presented the most comprehensive protocol to date with useful practical tips, spanning from surgical resection to long-term culture (Bak et al., 2024; see Supplementary Table 1). Using human CSF, they reported robust slice viability, efficient adeno-associated virus (AAV) transduction within 24 h, and stable patch-clamp recordings, validated with Streptavidin-based cell labeling (Bak et al., 2024).

Taken together, these studies have converged on several best practices: maintaining slices at 300–350 μm on membrane inserts, using human CSF or CSF-like media, and applying protocols that support survival for up to 2–3 weeks, with optimized systems extending viability to 6 weeks. While the viable culturing period is shorter than demonstrated for rodent OBSCs, human slices uniquely preserve patient-specific cellular diversity, cytoarchitecture, and circuit-level properties (Schwarz et al., 2019; Bak et al., 2024; Joseph et al., 2025), making them an invaluable model system. Continued refinement and cross-laboratory standardization will be essential to maximize their reproducibility and to expand their role in modeling human-specific mechanisms of health and disease.

### 2.2 Further developments and technical innovations

Recent advances have further improved the quality of human OBSCs. For instance, the permanent perfusion of slice cultures with a peristaltic pump ensures continuous nutrient and oxygen delivery while also removing metabolic waste. This approach reduced cell death by nearly 30% after 14 days in culture and improved electrophysiological integrity compared with conventional static methods (Romero-Leguizamón et al., 2019). Such technical refinements represent a significant step toward increased long-term viability and functional relevance of human OBSCs.

In parallel, human brain organoids have emerged as a powerful approach, particularly for studying the very early stages of human brain development, where access to tissue is extremely limited. Nonetheless, the majority of current brain organoid protocols remain limited to modeling early developmental stages (first half of gestation), frequently lack intrinsic microglial and vascular compartments, and suffer from hypoxic core formation, which leads to central necrosis (Urrestizala-Arenaza et al., 2024). Innovative strategies are now being explored to address these shortcomings. Notably, xeno-transplantation of organoids (as reviewed in Wang et al., 2023), co-culturing of human organoids with organotypic brain slice cultures (Panagiotakopoulou et al., 2025), or slicing organoids and culturing them as organotypic slices enhances oxygen and nutrient diffusion, sustains cell proliferation, and enhances development phenotype of the human organoids (Qian et al., 2020).

The establishment of more reliable culture conditions has enabled researchers to focus on functional interrogations through live-cell labeling approaches.

## 3 Live-cell physiology in human OBSCs

### 3.1 Live-cell labeling in human OBSCs

**Figure 2:**
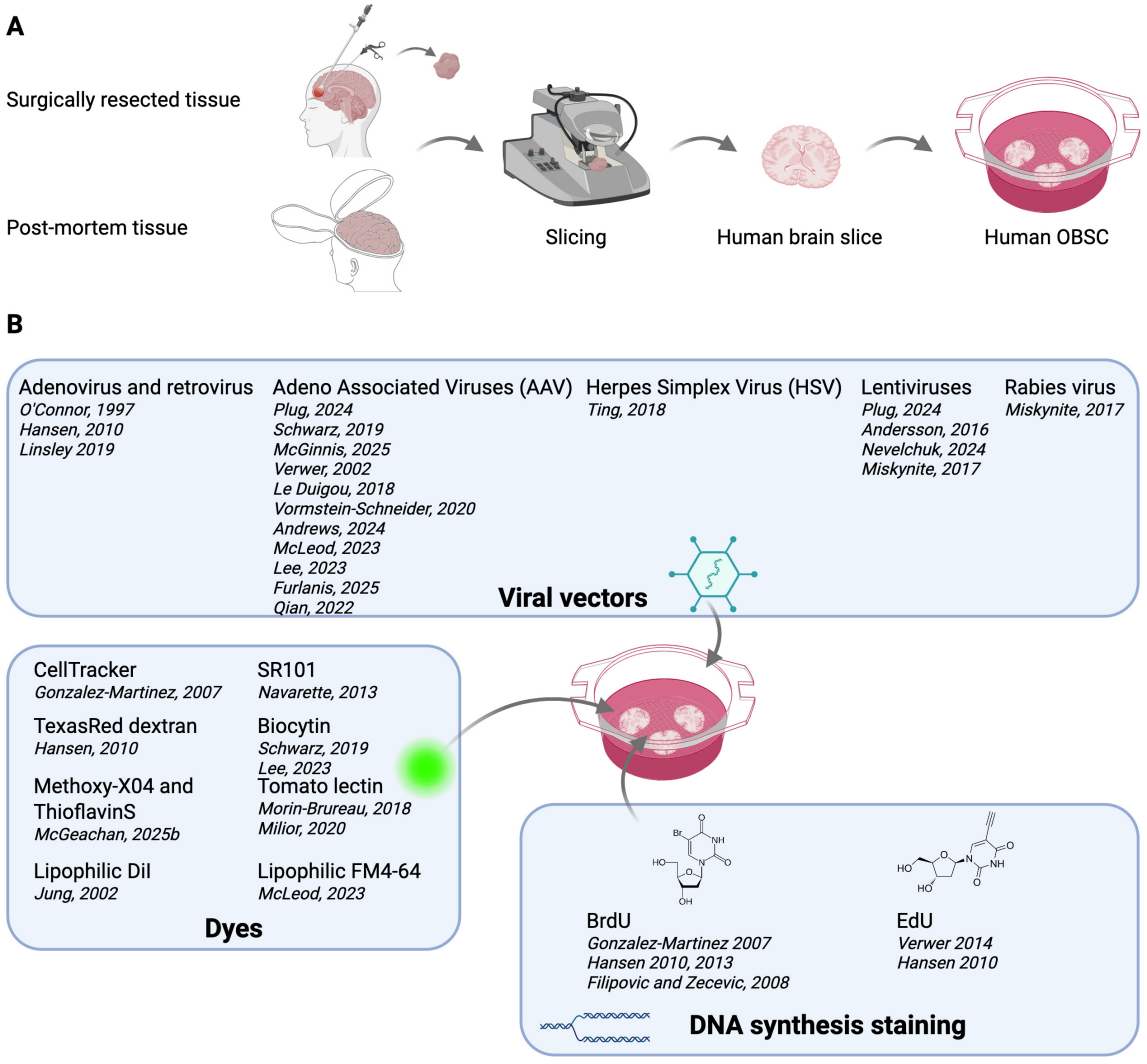
Preparation and live-cell labeling methods of human organotypic brain slice cultures. **(A)** Human tissue with surgery or *post-mortem* origin is sliced and cultured on membrane inserts in human CSF or CSF-like medium for 14–21 days. **(B)** Various currents methods used on human OBSC to live label cells in human OBSC involving viral vectors, dyes and BrdU/EdU. Created with BioRender.com.

The ability to label and manipulate cells in living human OBSCs has transformed their utility, enabling mechanistic insights into human brain physiology, pathology, and therapeutic interventions. Supplementary Table 1 provides an overview of key strategies and methods mentioned in studies using human OBSC. Below, we outline major approaches, emphasizing methodological innovations and their translational significance (Figure 2).

#### 3.1.1 Post-mortem human OBSCs: beyond clinical limitations

Several groups, including Dick F. Swaab's team in the Netherlands, pioneered the methods for culturing of postmortem human OBSCs obtained within 8 h of death (Verwer et al., 2002; Figure 2A). This strategy offered unique advantages: larger tissue availability, selection of defined brain regions, and access to non-pathological aged material. Verwer et al. (2002) used free floating human OBSCs from autopsies of healthy individuals and patients with Alzheimer's disease (AD), Lewy body (LB) dementia, multiple sclerosis (MS), and other forms of dementia. By infecting slices with recombinant AAVs carrying *Lac*Z, neurons and glia could be labeled and maintained for up to 78 days, demonstrating the feasibility of long-term gene expression in human brain tissue (Figure 2B). Their work, extending over two decades (Qi et al., 2019), established post-mortem OBSCs as a powerful model for studying the human brain. More recent studies expanded this platform to model white matter injury and demyelination. For, example, Plug et al. (2024) applied lysolecithin as demyelination model to post-mortem OBSCs, cultured them in human CSF, and successfully delivered viral vectors expressing green fluorescent protein (GFP), primarily targeting oligodendrocytes and microglia (Plug et al., 2024; Supplementary Table 1). This approach provides an unprecedented opportunity to combine human gene therapy strategies with a demyelination model in a human cellular context.

#### 3.1.2 Surgical resections: bridging pathology and therapeutic innovation

Fresh surgical resections provide access to younger, viable tissue (Figure 2A). Notwithstanding the ongoing debate surrounding the classification of such material as “healthy,” it nevertheless offers unique opportunities for investigation. O'Connor et al. (1997) demonstrated the feasibility of viral gene transfer by directly injecting Adenoviral vectors (Ad5) into resected tissue of epileptic patients prior to slicing, providing a landmark proof of concept for localized gene therapy in human brain tissue (O'Connor et al., 1997; Figure 2B, Supplementary Table 1). Resected OBSCs have also been used to interrogate neurogenesis in epileptic human tissue revealing, using bromodeoxyuridine (BrdU) labeling, migratory routes of newborn neurons not detectable in control tissue (González-Martínez et al., 2007). In fetal tissue studies, Kriegstein's group, elegantly used BrdU/EdU labeling, viral reporters, and electroporation to define the role of outer radial glia (oRG) in human cortical expansion (Hansen et al., 2010, 2013; Figure 2B, Supplementary Table 1). These studies underscore the translational potential of resected OBSCs for both basic developmental neurobiology and disease modeling. Linsley et al. (2019) subsequently asked whether it would be possible to track cell dynamics in human fetal brain slice culture over a period of 3 weeks. To address this, they infected fetal human slices with adenovirus CMV-GFP and performed live imaging using a custom-built automated confocal microscope and culture system. They were able to visualize the migratory pathways of cells along radial glia to study neocortical development, although the interpretation of the resulting images was not always straightforward (Linsley et al., 2019).

#### 3.1.3 Modern viral tools and live imaging: precision labeling and functional insights

Innovations in viral vectors and imaging techniques have further transformed the labeling landscape. Koch and Wuttke's teams optimized slice culturing in human CSF and achieved robust GFP labeling of neurons via AAV retrograde infection, enabling monitoring of spine dynamics using two-photon microscopy with remarkable stability (Schwarz et al., 2019; Figure 2B, Supplementary Table 1). McGinnis et al. (2024, 2025) extended this by integrating single-nucleus RNA sequencing (snRNA-seq) with viral infection to map AAV tropism across human cell types (McGinnis et al., 2024, 2025). They were able to map the most effective viruses for each cell type in human tissue by combining immunofluorescence and snRNA-seq of the infected cells. Overall, PHPs and PHP.eB showed the highest efficiency in infecting the largest number of cells, with astrocytes consistently displaying the best infection rates regardless of the viral serotype. Interestingly, PHPs performed poorly in mice. These results highlight the potential of human OBSC validation for gene therapy pipelines (McGinnis et al., 2025). In the following paragraphs, a more detailed discussion of cell-specific labeling approaches will be presented.

### 3.2 Neuron-specific labeling

**Figure 3:**
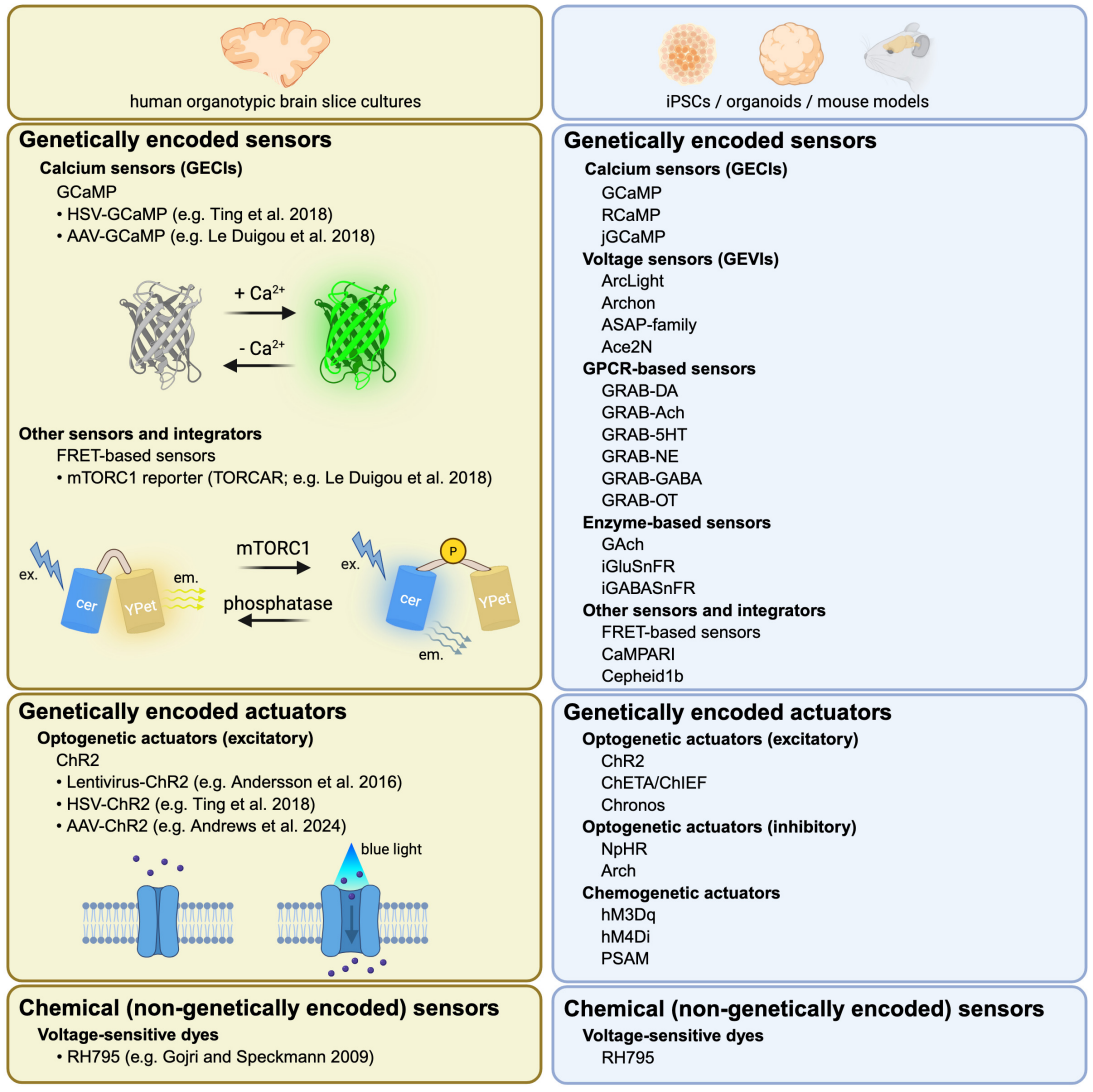
Cell-specific labeling tools in mouse and human OBSC and promising approaches. Neurons and subtypes have been widely targeted in mouse and human OBSC. In human OBSC, specific neurons could be targeted with specific promoters, enhancers or miRNAs. Astrocyte labeling in human OBSC has only been reported with dyes although specific viruses exist. Microglia remain challenging to target with viruses but some efforts have shown conclusive results with dyes and viruses. Finally, more work needs to be done for the study of oligodendrocytes in human tissues. Created with BioRender.com.

Besides serotype selection, promoter choice provides a key layer of specificity for targeted gene expression in OBSCs. Neuronal promoters such as human synapsin I (hSyn) and CaMKIIα have been widely used to drive expression in excitatory neurons (Kügler et al., 2003; Nathanson et al., 2009), whereas Dlx enhancer elements selectively target GABAergic interneurons (Dimidschstein et al., 2016; Lee et al., 2023; Figure 3, Supplementary Table 1). These promoter systems have been extensively validated in rodent OBSCs and *in vivo*, where they achieve robust, subtype-specific transduction across cortical and hippocampal regions (Furlanis et al., 2025). Dimidschstein's team further identified *Scn1a* enhancers that specifically target PV interneurons and demonstrated enhanced viral expression in human OBSCs (Vormstein-Schneider et al., 2020). Using Patch-sequencing in human OBSCs, Lee et al. (2023) generated a detailed transcriptomic map of GABAergic neurons based on their morphology and electrophysiological properties (Lee et al., 2023). This map could be used to tailor promoters or enhancers for targeting specific neuronal subsets. Another study reported the use of scATAC-seq to identify enhancers in GABAergic interneurons and described the VIP neuronal subtype enhancer BiVIPe4, which was used to virally deliver ChR2 and mCherry into human OBSC (Furlanis et al., 2025). Recently, Wirthlin et al. (2026) generated a library of enhancer-driven AAVs to target and manipulate neurons in the mouse basal ganglia, including enhancers with human orthologs. Future studies in human OBSCs should systematically assess promoter–serotype combinations, particularly with AAV variants showing improved human tropism, such as AAV2, AAV5, and AAV9 (McGinnis et al., 2025), to refine targeting precision and support both experimental and therapeutic applications.

Further viral specificity can be achieved by coupling promoters with microRNA-dependent regulation or enhancer-based targeting strategies (Geisler and Fechner, 2016; Nevelchuk et al., 2024; Potenza et al., 2024; Figure 3). Recent technologies like CellREADR applied RNA sensing with ADAR to target specific RNA expressing cells, for instance *FOXP2* expressing neurons, and deliver an mNeon reporter in human OBSC (Qian et al., 2022). These modular approaches are expected to enhance cell-type and even layer-specific expression in human tissue, advancing both basic research and translational gene modulation platforms.

### 3.3 Labeling glial populations

#### 3.3.1 Astrocyte labeling

Astrocytes have been infrequently targeted in human OBSCs (Figure 3). Navarrete et al. (2013) first demonstrated bidirectional signaling between human neurons and astrocytes using Fluo-4 AM calcium imaging and SR101 labeling in acute human slices from epileptic patients, revealing slow astrocytic calcium waves and activity-dependent neuron-astrocyte coupling (Navarrete et al., 2013; Supplementary Table 1). In mouse OBSCs, astrocytic calcium imaging using GCaMP indicators under the *GFAP* or *Aldh1l1* promoters has become standard practice (Shigetomi et al., 2013; Haustein et al., 2014; Srinivasan et al., 2016; Koh et al., 2017). New tools emerged to increase astrocyte specificity for instance by inserting microRNA to decrease drastically non-specific neuronal expression (Gleichman et al., 2023; Figure 3). These approaches, together with AAV vectors optimized for glial transduction (e.g., AAV5, AAV8, AAV9), are highly adaptable to human tissue and would enable direct investigation of gliotransmission, neurovascular coupling, and astrocytic modulation of neuronal activity.

#### 3.3.2 Microglia labeling

Microglia remain challenging to label or transduce efficiently in human slices (Figure 3). Using tomato lectin staining in acute epileptic tissue, Morin-Brureau et al. (2018) and Milior et al. (2019, 2020) characterized distinct microglial phenotypes: reactive amoeboid cells lacking purinergic responsiveness, and homeostatic ramified microglia capable of rapid process retraction and extension upon ADP stimulation (Morin-Brureau et al., 2018; Milior et al., 2019, 2020; Supplementary Table 1). Of note, another fluorescent lectin, Isolectin B4, has been used in mouse OBSCs to live label microglia (Dailey et al., 2013).

Recently, Nevelchuk et al. (2024) introduced a major technical advance by recording microglial calcium signaling in human OBSCs (Figure 3, Supplementary Table 1). They used a lentiviral construct containing microRNA (miR9) target sequences to restrict expression to microglia, allowing specific expression of CaNeon, a low-interference, Troponin-C-based calcium sensor fused to mNeon green. This design includes only two calcium-binding domains instead of four, as seen in GCaMP, thus minimizing interference with physiological calcium pathways. This innovative approach enabled long-term imaging of calcium transients in >80% of IBA1-positive cells and processes compartments, revealing higher calcium activity in ramified compared to amoeboid microglia (Nevelchuk et al., 2024).

Insights from mouse OBSCs illustrate the dynamic sensitivity of microglia to slicing and culture conditions. Immediately after slicing, microglia migrate toward cut surfaces, lose homeostatic markers such as P2Y12 and P2Y12R, and transiently adopt an activated phenotype (Czapiga and Colton, 1999; Berki et al., 2023). Consistent with these observations, the microglial inflammatory profile has been shown to increase during the first 1–2 weeks *in vitro*, marked by elevated expression of activation-related genes such as CD68 and CD44, before stabilizing. By 3 weeks, microglia recover transcriptomic and morphological hallmarks of mature, *in vivo*-like cells. However, prolonged culture can induce neurodegeneration-associated gene signatures (Delbridge et al., 2020).

Donor-specific factors, including age and disease state, further modulate microglial baseline activation, as shown in slices from post-mortem tissue derived from patients with metachromatic leukodystrophy exhibiting pre-existing microglial activation characterized by enlarged, rounded morphologies and altered lysosomal profiles (Plug et al., 2024).

#### 3.3.3 Oligodendrocyte labeling

Few studies have focused on oligodendrocytes in human OBSCs (Figure 3). Jakovcevski et al. (2007) studied myelination in fetal OBSCs. Using immunostainings, they demonstrated that PSA-NCAM, a cell adhesion molecule, was predominantly expressed in neurons, in contrast to findings in mice where it is primarily found in glial cells. Furthermore, they noted that after 5 days *in vitro*, the expression of PSA-NCAM decreased, allowing myelination to occur along with an increase in myelin basic protein (MBP) signal (Jakovcevski et al., 2007). Later work from the same group explored CXCL1-driven oligodendrocyte progenitors cells (OPCs) proliferation in fetal OBSCs using BrdU and TUNEL assay to quantify proliferation and apoptosis (Filipovic and Zecevic, 2008; see Supplementary Table 1). These studies highlight that oligodendroglial development and myelination proceed *in vitro*, providing a basis for studying human myelin plasticity and pathology. In rodent slices, the MBP, 2′3′-cyclic nucleotide 3′-phosphodiesterase (CNPase), and myelin oligodendrocyte glycoprotein (MOG) promoters reliably target oligodendrocytes, whereas PDGFRα promoters or enhancer elements selectively target OPCs (Yuan et al., 2002; von Jonquieres et al., 2013, 2016; O'Rourke et al., 2016). Other teams used fusion protein delivered by viruses to target specifically oligodendrocytes for instance with mCherry-fused MBP with Semliki Forest Virus (SFV; Toth et al., 2021). Selective dyes such as Fluoromyelin have also been used on the living mouse brain to label myelin although it can only be used acutely (Kamen et al., 2025). Interestingly, the same team used the dye SR101 to label oligodendrocytes thanks to gap-junction connections between astrocytes and oligodendrocytes. These tools provide a roadmap for translating these strategies to human tissue to investigate activity-dependent myelination and remyelination processes.

#### 3.3.4 Best practices and lessons for live cell labeling of human OBSC

On a broader scale, standardized protocols for human OBSC live cell-labeling remain largely absent. Currently, many laboratories develop their own protocols independently. A coordinated effort through the establishment of dedicated working groups or consortia focused on human OBSCs would facilitate protocol standardization, improve robustness and reproducibility, and ensure the most effective use of this precious tissue.

However, several key points have emerged that indicate best practices supported by multiple laboratories and recent data (Supplementary Table 1). In particular, viral strategies employing AAV infection to deliver reporters have emerged as a powerful and widely adopted approach:

- *Serotypes and promoters:* Recent data from McGinnis et al. (2025) suggest that the PHP.eB serotype enables broad expression in human OBSCs, although further empirical testing is required for specific cell types. Several laboratories have reported successful neuronal targeting using the AAV2 retrograde serotype (Schwarz et al., 2019; Qian et al., 2022), the PHP.eB serotype (Vormstein-Schneider et al., 2020; Plug et al., 2024; Furlanis et al., 2025), or neuronal promoters such as the Synapsin promoter (Andersson et al., 2016; Le Duigou et al., 2018; Qian et al., 2018; Ting et al., 2018; Schwarz et al., 2019; Eggert et al., 2026). For other cell types, including astrocytes and oligodendrocytes, insufficient data are currently available to identify a preferred strategy. For microglia, Tomato Lectin injections have been reported as an effective labeling approach when the methodology is carefully optimized (Morin-Brureau et al., 2018; Milior et al., 2020).
- *Timing of infection*: A strong consensus across studies indicates that the optimal time for viral infection is either on the day of slice preparation or the following day to allow tissue recovery (O'Connor et al., 1997; Andersson et al., 2016; Le Duigou et al., 2018; Ting et al., 2018; Linsley et al., 2019; Vormstein-Schneider et al., 2020; Lee et al., 2023; McLeod et al., 2023; Andrews et al., 2024; Nevelchuk et al., 2024; Furlanis et al., 2025; McGinnis et al., 2025).
- *Viral titer:* Considerable variability exists in the titers used across studies. High viral titers are frequently applied in undiluted form from production or the manufacturer. Reported AAV titers typically range between 10^9^ and 10^12^ Vg/mL (Le Duigou et al., 2018; Ting et al., 2018; Schwarz et al., 2019; Table 1), although these parameters should be optimized empirically for each experimental setup. In many protocols, approximately the virus is applied vertically to the center of the slice, where it remains on the surface of the slice through surface tension.
- *Duration of expression:* Viral constructs typically show expression between seven and 14 days (Supplementary Table 1).

**Table 1:** Viral strategies comparison between herpes simplex virus (HSP), adeno-associated virus (AAV), and lentivirus.

Feature	HSV	AAV	Lentivirus
Payload capacity	Up to 150 kb	~4.7 kb	~8–10 kb
Titers used for human OBSC (Supplementary Table 1)	~10^9^ infecting units/mL	10^7^-10^12^ Vg/mL	10^6^-10^11^ viral particles/mL
Transduction efficiency	Excellent in neurons (fast, widespread)	Good, varies by serotype	Good (esp. in dividing & non-dividing cells)
Tropism	Strong natural neuron tropism	Serotype-dependent	Broad; pseudotyping allows CNS targeting
Duration of expression	Moderate–long (episomal, not stable)	Long-term (episomal in non-dividing cells)	Long-term, stable integration into genome
Genome integration	No	No (mostly episomal)	Yes (random integration)
Immunogenicity	Mild–moderate (less than wild HSV)	Very low	Moderate (depends on envelope, promoter)
Onset of expression	Fast (hours−1 day)	Slower (~2–5 days)	Moderate (~2–3 days)
Production difficulty	Complex (needs helper virus or special cell lines)	Well-established protocols, scalable	Standard triple-transfection in 293T cells
Ideal for	Large constructs, BACs, rapid neuronal expression	Long-term expression, systemic delivery	Stable integration, knockdown, CRISPR, moderate size
Biosafety levels (EU)	BSL-2	BSL-1/2	BSL-2

The development of these live-cell labeling approaches in human OBSCs has enabled functional interrogation of living cells. The following section discusses these applications in greater detail.

## 4 Functional manipulations of living human OBSCs

### 4.1 Functional manipulation and optical readouts in human OBSCs

#### 4.1.1 Optogenetics and calcium imaging

**Figure 4:**
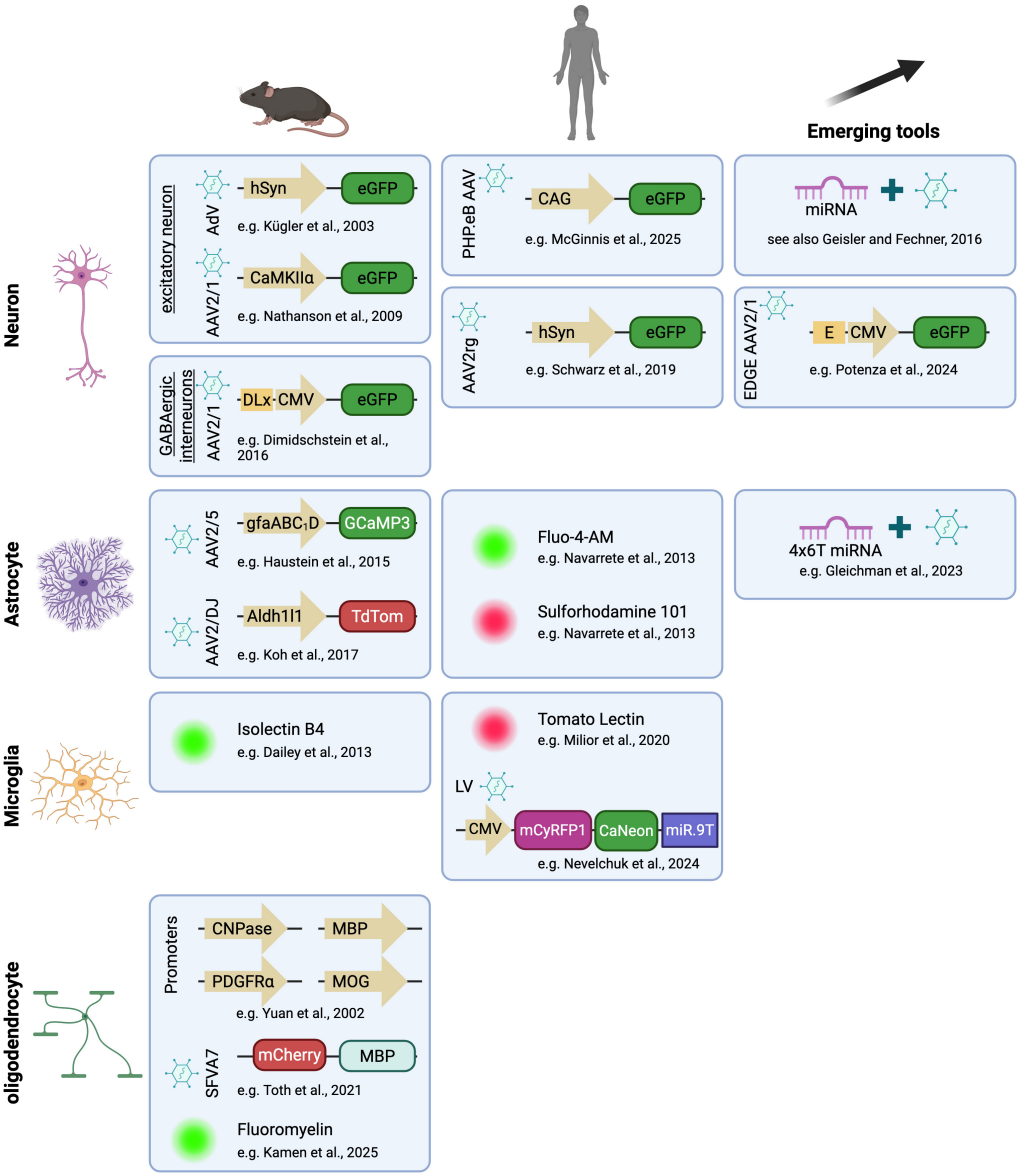
Cell-specific labeling tools in mouse and human OBSC and promising approaches. Neurons and subtypes have been widely targeted in mouse and human OBSC. In human OSC, specific neurons could be targeted with specific promoters, enhances or miRNA. Astrocyte labeling in human OBSC has only been reported with dyes although specific viruses exist. Microglia remain challenging to target with viruses but some efforts have shown conclusive results with dyes and viruses. Finally, more work needs to be done for the study of oligodendrocytes in human tissues. Created with BioRender.com.

Optical tools allow functional readouts and manipulation of neuronal activity in human OBSCs (Figure 4). Optogenetic control in human OBSCs was first demonstrated by Andersson et al. (2016), who demonstrated reliable light-evoked action potentials following lentiviral Channelrhodopsin-2 (ChR2) expression (Andersson et al., 2016). Subsequent work by Ting et al. (2018) extended this approach using herpes simplex virus type 1 (HSV-1) vectors to deliver opsins, calcium sensors, and reporters for combined activity monitoring and modulation (Figures 2B, 4, See Table 1 for viral comparisons; Ting et al., 2018). Genetically Encoded Calcium Indicators (GECIs) are calcium binding proteins coupled to fluorescent reporters. Among the most widely used are GCaMP indicators, which consist of a fusion between the calcium-sensitive Calmodulin (CaM) and a circularly permutated variant of green fluorescent protein (GFP). Upon binding calcium ions (Ca^2+^), CaM undergoes a conformational change that restores the fluorescent properties of GFP, enabling optical detection of neuronal activity. For example, Le Duigou et al. (2018) used AAV-mediated expression of GCaMP to record epileptiform activity in patient-derived slices in combination with electrophysiological recordings (Le Duigou et al., 2018). Other functional approaches include the use of *Scn1a* enhancer-driven AAVs to target parvalbumin (PV) interneurons and deliver the red shifted opsin C1V1 in human OBSC, which successfully increased neuronal firing rates upon photoactivation (Vormstein-Schneider et al., 2020). In another approach, magnetoelectric nanoparticles (MENPs) were injected to stimulate neuronal activation using an alternating magnetic field, thereby increasing calcium signaling in GCaMP-expressing neurons (Eggert et al., 2026). Recently, Andrews et al. (2024) achieved real-time optogenetic suppression of seizure-like discharges in human OBSCs, illustrating the potential of these systems for personalized neuromodulation in human tissue (Andrews et al., 2024; Supplementary Table 1).

It is important to note that calcium imaging results are greatly influenced by both the region and the age of the patient, with the most robust results typically obtained from hippocampal slices or fetal brain tissue [unpublished observations and (Eugène et al., 2014; Le Duigou et al., 2018)]. In addition, calcium imaging, unlike in human OBSCs, is routinely performed in mouse OBSCs. Mouse OBSCs, which are typically derived from early postnatal pups (approximately 4–7 days old), show higher AAV-mediated transduction efficiency and more consistent labeling compared with human slices (unpublished observations). These highly efficient network-scale recordings and manipulations can serve as a blueprint for similar applications in human slices.

Red-shifted GECIs (e.g., REX-GECO1; Wu et al., 2014) further improve imaging depth and could facilitate studies in thicker human brain tissue slices. Comparable approaches have been widely implemented in human iPSC-derived brain organoids, where calcium imaging has revealed developmental maturation and pharmacological responsiveness (Sakaguchi et al., 2019; Park et al., 2024). Recent advances, such as the bicistronic mRuby2-GCaMP6s construct (He et al., 2025), enhance co-expression efficiency and imaging stability, underscoring strategies that may translate to human slice models. Improved variants such as GCaMP-X (Yang et al., 2018; Geng et al., 2022), tested for mouse primary neurons and mouse cortex OBSC, minimize cytotoxicity and are particularly relevant for long-term imaging in human OBSCs.

Beyond optogenetics, chemogenetic actuators such as Designer Receptors Exclusively Activated by Designer Drugs (DREADDs) offer a complementary approach for targeted modulation in human slice cultures if sufficient viral tropism can be achieved (Mueller et al., 2022). Recently, pharmacologically selective actuator modules (PSAM) have improved selectivity of receptor targets with very low endogenous ligand binding in mouse OBSCs, mice and rhesus monkey, suitable for future human research and drug development for therapies (Magnus et al., 2019; Gonzalez-Ramos et al., 2025).

#### 4.1.2 Voltage and signaling reporters

Voltage-sensitive dyes were among the first tools used to monitor network dynamics in human epileptic slices (Gojri and Speckmann, 2009), revealing burst activity through fast optical recordings. However, these dyes are limited by phototoxicity and poor cell-type specificity. Genetically encoded voltage indicators (GEVIs) overcome some of these constraints, offering millisecond temporal resolution and targeted expression through viral vectors. However, their relatively low signal-to-noise ratio and the potential toxicity remain significant challenges. In mouse OBSCs, fluorescent voltage indicators have already gained increasing popularity. While GECIs generally provide higher signal-to-noise ratios, GEVIs offer superior temporal resolution, enabling the detection of rapid membrane potential changes and more precise discrimination of neuronal population activity (Zhu et al., 2021), as demonstrated in mouse OBSCs with Archon1 and Cepheid1b (Piatkevich et al., 2018; Han et al., 2023). Other established GEVI families, such as ASAP and QuasAr, also provide valuable alternatives for high-speed voltage imaging in slice preparations (Adam et al., 2019; Villette et al., 2019). Ongoing improvements in two-photon GEVI imaging and expression stability make these tools promising candidates for future human applications.

Finally, fluorescent reporters extend functional imaging beyond membrane dynamics. Le Duigou et al. (2018) employed a FRET-based mTORC1 reporter (TORCAR) in human OBSCs, revealing preserved signaling homeostasis during seizures (Le Duigou et al., 2018). Such studies illustrate how integrated optical, electrophysiological, and molecular approaches can transform human slice cultures into powerful experimental platforms for probing circuit function and intracellular signaling in native human brain tissue.

#### 4.1.3 Next generation functional reporters and biosensors

Beyond calcium and voltage indicators, a new generation of genetically encoded biosensors enables optical readouts of neurotransmitter and metabolic signaling in living brain tissue. These sensors, based on conformational changes of G-protein coupled receptors (GPCRs) or fluorescent protein domains, can be expressed via viral delivery and targeted to specific cell types using appropriate promoters or regulatory elements. In rodent OBSCs and organoids, such sensors have been integrated with optogenetic and calcium imaging systems, enabling multiplexed monitoring of neuronal and glial signaling. Examples include acetylcholine biosensors (GACh; Jing et al., 2018); dopamine sensors (gGRAB and dLight; Dong et al., 2025); serotonin sensors (sDarken; Kubitschke et al., 2022); GABA sensors (iGABASnFRs; Kolb et al., 2025); and glutamate sensors (iGluSnFRs; Aggarwal et al., 2023). Metabolic receptors such as LiLac and FiLa track glucose fluxes (Li et al., 2024), while G-protein-coupled receptor activation (GRAB)-based designs enable detection of neuromodulator release, including norepinephrine (GRAB_NE_; Feng et al., 2024) and oxytocin (GRAB_OT_; Qian et al., 2023).

In conclusion, calcium imaging (using virally delivered GECIs), voltage imaging (using synthetic dyes), and optogenetics (using virally delivered ChR2) have been applied to human OBSCs, but progress has been constrained in part by low viral transduction efficiency. Future studies could benefit from incorporating approaches that are well-established in mouse models and human iPSC-derived systems, including chemogenetics, GEVIs, and additional classes of biosensors.

### 4.2 Functional manipulation, cell replacement, and molecular dissection in human OBSCs

Human OBSCs provide a unique experimental platform that bridges the gap between *in vitro* systems and the human brain's native cytoarchitecture. Beyond serving as a model for physiological network activity, OBSCs enable diverse manipulations, from grafting and depletion to gene editing and omics profiling, offering unprecedented opportunities to study human neurodevelopment, disease mechanisms, and regenerative strategies within an intact microenvironment.

#### 4.2.1 Grafting cells and integration in human OBSCs

The grafting of cells in human OBSCs facilitates the investigation of cell-cell interactions, migration, and synaptic integration within a preserved human microenvironment. Early studies primarily focused on tumor invasion. For example, Jung et al. (1999, 2002) pioneered the use of human OBSCs to investigate astrocytoma and glioma infiltration, revealing that extracellular matrix components such as elastin enhanced astrocytoma cell invasion. More recently, Ravi et al. (2019), Czolk et al. (2019), Maier et al. (2021), Schneider et al. (2021), Ravi et al. (2022), and Buehler et al. (2023) refined these models using patient-derived glioblastoma cells expressing fluorescent reporters or CRISPR-modified variants to visualize invasion dynamics, drug sensitivity (to Temozolomide or Meclofenamate for instance), molecular drivers such as FBXO2 or GRM3, and multi-omics studies (Czolk et al., 2019; Ravi et al., 2019, 2022; Maier et al., 2021; Schneider et al., 2021; Buehler et al., 2023; Supplementary Table 1). In addition, to assess the connectivity of glioblastoma cells with surrounding neurons, researchers have introduced a new tracing tool based on rabies viruses and modified glioblastoma cells able to propagate the virus to connected cells (Tetzlaff et al., 2025). Their findings highlighted the potential of OBSCs as an *ex vivo* human platform for therapeutic testing and precision oncology.

Human OBSCs have also been applied to study neuronal replacement and graft integration. Miskinyte et al. (2017) transplanted human fibroblast-derived neurons into cortical slices and combined rabies-virus tracing and functional imaging to confirm host-graft synapse formation (Miskinyte et al., 2017). Building on this work, Grønning Hansen et al. (2020) grafted human induced pluripotent stem cell (iPSC)-derived long-term neuroepithelial-like stem cells (lt-NES) into human slices, revealing reciprocal afferent and efferent connectivity with host neurons (Grønning Hansen et al., 2020). Although the grafted neurons exhibited more immature electrophysiological properties (Grønning Hansen et al., 2020), these studies demonstrated that iPSC-derived neurons can structurally and functionally integrate into human brain tissue *ex vivo*, supporting future cell replacement therapies for neurodegenerative disorders.

In a developmental context, recent work from the Kriegstein's group identified a novel progenitor population (Tri-IPCs), capable of generating astrocytes, OPCs, and interneurons (Wang et al., 2025). Using adenoviral CMV-GFP labeling and grafting into fetal human OBSCs, they validated the multipotent lineage potential predicted from single-cell transcriptomics. Together, these grafting studies underscore OBSCs as a flexible platform for studying cell migration, synaptogenesis, and graft–host connectivity across both physiological and pathological contexts.

Despite their potential, challenges remain. Resident microglia and astrocytes can mount localized inflammatory responses following grafting or slicing, which may impair graft maturation and functional integration (Delbridge et al., 2020). Moreover, culture longevity in human tissue is typically limited to 2–3 weeks due to metabolic and oxygenation constraints (Humpel, 2015). Future developments may involve transient immune modulation, optimized oxygenation interfaces, or co-culture with vascular or endothelial components to sustain long-term integration and maturation (Qian et al., 2018; Cakir et al., 2019).

#### 4.2.2 Cell depletion and functional dissection

While labeling and grafting have been widely adopted, targeted depletion approaches in human OBSCs remain rare but informative. Filipovic and Zecevic (2008) selectively ablated astrocytes and microglia in human fetal OBSCs using alpha-amino-adipic acid (alpha-AAA) and L-leucine-methyl-ester (L-LME), respectively. This revealed astrocytes as the principal source of CXCL1, which acts via CXCR2 signaling on both astrocytes and OPCs to drive proliferation through ERK1/2 and IL-6 pathways (Filipovic and Zecevic, 2008; Supplementary Table 1).

Although these chemical methods are limited by off-target effects and transient depletion, they illustrate the potential of targeted ablation in human OBSCs to dissect cell-type-specific contributions in complex human networks. Future genetic or inducible strategies, such as Caspase-9-based “suicide switches” (Klopp et al., 2021) or cell-type-restricted diphtheria toxin receptor (DTR) expression under inducible or Cre-dependent promoters (Buch et al., 2005), could refine depletion approaches with higher precision and temporal control.

In human OBSCs, such systems could be implemented via AAV- or lentiviral vectors carrying inducible constructs driven by cell-specific promoters. Coupling these tools with fluorescent reporters or biosensors would permit live tracking of degeneration, circuit remodeling, and glial activation in response to defined cell-type loss. Such strategies, already validated in rodent and stem-cell–derived systems, hold promise for establishing causal relationships between specific human cell populations and functional network outcomes within the native microenvironment of OBSCs.

#### 4.2.3 Pharmacological and genetic manipulation

Pharmacological modulation remains a straightforward and widely used approach to probe signaling pathways in human OBSCs. Using human fetal slices, Lui et al. (2014) demonstrated that PDGFRβ signaling regulates radial glial proliferation, an effect absent in E13.5 mouse cultures, revealing species-specific growth factor dependencies (Lui et al., 2014). Similarly, Wang et al. (2025) investigated somatostatin (SST) receptor activation in early human cortical development, showing that SST receptor agonists suppress synaptogenesis and neurite extension while enhancing metabolic gene expression in excitatory neurons (Wang et al., 2025). These findings emphasize the potential of OBSCs to analyze neurodevelopment, capturing human-specific signaling that is absent in rodents.

The same group investigated nicotinic acetylcholine receptor (nAChR) signaling using fetal OBSC exposed to nicotine, finding that chronic exposure enhances radial glial proliferation through nAChR-dependent mechanisms (Mukhtar et al., 2025). Lentiviral shRNA-mediated receptor knockdown reversed this phenotype, indicating direct causal involvement and highlighting the model's utility for toxicology and developmental neurobiology. Such approaches could readily be extended to investigate the impact of environmental factors, drugs, pesticides, heavy metals, or microplastics, on neurogenesis and circuit maturation.

Adam et al. (2026) investigated neuroinflammation and cell-type specific pathways in long-term human OBSCs following TNFα treatment. Utilizing a combination of bulk and single-nucleus RNA sequencing, the authors identified significant alterations in glial cell populations following treatments involving pro- and anti- inflammatory programs. These data facilitate interpreting human cell-specific responses in neuroinflammatory contexts, such as neurodegenerative diseases, while also emphasizing the multifaceted roles of glial cells.

#### 4.2.4 Emerging gene editing and molecular profiling tools

CRISPR/Cas9-based technologies represent the next frontier for genetic manipulation in OBSCs. Compact Cas9 variants such as SaCas9 have been packaged in AAV vectors to achieve efficient genome editing in neurons (Deutsch et al., 2021), and CRISPR-based transcriptional regulators such as dCas9–VPR or dCas9–KRAB allow reversible activation (CRISPRa) or repression (CRISPRi) of endogenous genes without altering the genome. When delivered via dual AAV or lentiviral systems, these constructs can target specific neuronal or glial populations using promoter-driven or enhancer-restricted expression. Such strategies have been used in mouse organotypic slices to dissect ion channelopathies (Tsortouktzidis et al., 2022), and could be extended to human OBSCs to manipulate disease-relevant pathways or to test rescue of pathogenic variants identified in patient-derived tissue. Integration with activity reporters or opto/chemogenetic effectors (e.g., hM3Dq, hM4Di, or ChrimsonR) could further allow real-time correlation of molecular perturbations with network-level dynamics, providing a powerful human-specific framework for causal functional genomics.

At the systems level, transcriptomic and proteomic profiling of human brain tissue has revealed profound interspecies differences in cellular composition and signaling (Siletti et al., 2023). Recent single-cell RNA sequencing (scRNA-seq) studies on acutely resected human tissue (Liharska et al., 2025) demonstrate marked gene-expression shifts compared with post-mortem samples, emphasizing the value of living tissue-based analyses. Future integration of OBSCs with single-cell omics, lipidomics (Osetrova et al., 2024), and proteomics (Tüshaus et al., 2023) could uncover dynamic molecular trajectories of human neuronal networks during culture or disease progression.

Together, these novel approaches to functionally dissect human brain physiology hold great promise to facilitate a more profound comprehension of human brain diseases which we will discuss briefly in the next part.

## 5 Modeling human disease and therapeutic testing

Human OBSCs can facilitate modeling disease processes and human-specific network dynamics in a 3D *ex vivo* brain tissue environment. In oncology, glioma invasion and therapeutic responses have been assessed using patient-derived tumor cells engrafted into human slices (Jung et al., 1999, 2002; Ravi et al., 2019, 2022; Buehler et al., 2023). In epilepsy research, Le Duigou et al. (2018) expressed the genetically encoded calcium indicator GCaMP via AAV vectors to monitor epileptiform activity (Le Duigou et al., 2018; Supplementary Table 1). Andrews et al. (2024) further achieved real-time optogenetic suppression of seizure-like discharges in patient-derived OBSCs, demonstrating the translational potential of such approaches for personalized neuromodulation (Andrews et al., 2024).

Neurodegenerative disease modeling has similarly benefited from this platform. Mendes et al. (2018) showed that amyloid beta (Aβ) Aβ1–42 exposure in a human tissue context induces tau phosphorylation and synaptic loss, reproducing early Alzheimer's disease (AD) pathology (Mendes et al., 2018). McGeachan et al. (2025a,b) extended these findings by dissecting Aβ and tau dynamics, revealing bidirectional regulation of amyloid processing by BACE1 and metalloprotease inhibition, as well as trans-synaptic spread of progressive supranuclear palsy (PSP)-derived tau oligomers (McGeachan et al., 2025a,b; Supplementary Table 1). Alpha-synucleinopathies have also been modeled in human OBSCs (Barth et al., 2021). By using AAV-mediated overexpression of alpha-synuclein in combination with pre-formed fibrils, the authors demonstrated the formation of alpha-synuclein inclusions in neurons, highlighting the potential for investigating Parkinson's disease mechanisms in human tissue (Barth et al., 2021). Such models bridge molecular pathology with functional outcomes in human neural circuits, facilitating preclinical testing in authentic tissue environments.

More recently, McLeod et al. (2023), used fetal human OBSCs to study the impact of gene variants for epileptic encephalopathies in brain development. The authors focused on Syntaxin-binding protein 1 (STXBP1), a synaptic protein associated with developmental encephalopathy when mutated. Their results showed that STXBP1 knockdown altered synaptic stability and confirmed a reduction in exocytosis (McLeod et al., 2023). This work provides a direct functional link between human disease gene variants and neuronal network alterations in a native tissue context.

Beyond classical neuropathologies, OBSCs are increasingly used to study viral neuropathogenesis, including SARS-CoV-2, herpes virus or Zika flavivirus infections, as summarized by Almeida et al. (2025). Combined with advanced imaging, biosensors, and high-content omics, human OBSCs have the potential to become a central platform for translational neuroscience.

## 6 Challenges and limitations

Despite the transformative potential of human OBSC, several limitations remain:

*Tissue accessibility:* Access to human brain tissue is limited and depends on close coordination among researchers, clinicians, and surgeons, as well as on informed patient consent. Consequently, each sample is both valuable, rare, and is associated with substantial biological variability.

*Tissue heterogeneity and logistical constraints:* The anatomical origin of resected tissue varies considerably. Most commonly, samples are obtained from the frontal or temporal cortex or the hippocampus. The underlying pathologies necessitating surgical intervention also differ, including various forms of epilepsy and brain tumors. Previous therapeutic interventions, such as chemotherapy or radiation, may further compromise tissue viability. In addition, surgical techniques vary between centers, including the use of surgical instruments, such as the Cavitron Ultrasonic Surgical Aspirator (CUSA), which may influence tissue integrity (Straehle et al., 2023). Transport logistics between the operation room and the lab also affect the tissue properties. While retaining most features, electrophysiological signatures and spine architecture are affected during transport (Qi et al., 2026). Oxygenation, hypothermia and anti-vibration transport boxes are solutions to reduce mechanical stress (Qi et al., 2026).

*Donor-to-donor variability:* The age of the patient has been demonstrated to exert a strong influence on the outcomes of tissue culture. For example fetal material, which exhibits high plasticity under culture conditions, tissue samples obtained from elderly patients, often present more difficult to maintain in culture. Additional factors such as ethnicity and sex may also contribute to biological variability. One solution to reduce variability is to use the same patient-derived tissue for both the control and experimental conditions.

*Laboratory standardization:* Differences in experimental protocols further complicate cross-study comparisons. For example, Schwarz et al. (2019) analyzed cortical tissue from patients aged 10–67 years undergoing surgery for intractable epilepsy, with samples obtained from the temporal or frontal lobe (Schwarz et al., 2019). In contrast, McGeachan et al. (2025a) studied tissue from patients aged 32–71 years undergoing surgery for glioblastoma, glioma or metastasis, with samples derived from the parietal, occipital, temporal or frontal lobes; ethnicity was not reported. These examples illustrate that the source of tissue can significantly diverge between centers and studies, limiting reproducibility. The use of human CSF as a culture medium can further introduce variability, as CSF composition differs between individuals. Given the inherent heterogeneity of patient-derived material, strategies such as pooling CSF samples or refining artificial culture media may help to reduce variability.

*Limited survival in culture:* While rodent slice cultures can survive for months, human OBSCs typically remain viable for only 2–3 weeks, partly due to donor maturity and increased metabolic stress. Improvements in oxygenation, media composition, and perfusion systems may help extend culture lifespan.

*Ethical considerations:* The use of fetal or post-mortem human tissue is subject to strict ethical considerations, including regulations surrounding consent for human samples, cerebrospinal fluid (CSF), and fetal material. These considerations have been discussed in detail by Joseph et al. (2025). In brief, dual consent is required for both clinical and research use, provided either by the patient or the next of kin. Additional regulatory frameworks include institutional ethics committee approval and donor privacy protections, such as de-identified samples and compliance with regulation including the General Data Protection Regulation (GDPR) in the European Union (EU) and the Health Insurance Portability and Accountability Act (HIPAA) in the United States of America (USA; Joseph et al., 2025).

Joseph et al. (2025) also discuss broader ethical questions that may emerge as human tissue culture technologies advance. These include whether isolated neural tissue capable of information processing could raise questions about consciousness, whether extended tissue survival in culture challenges current definitions of life and death, and whether emerging technologies such as gene editing combined with artificial intelligence could introduce biosafety concerns. Addressing such issues will require evolving international ethical frameworks and careful consideration of cultural and regulatory differences across countries

*Experimental variability:* Variability in viral transduction, slice preparation, and lipofuscin auto-fluorescence, especially in aged samples, poses challenges for imaging and quantification. Future progress will depend on standardized protocols, serotype–promoter mapping, and integration of reporter systems compatible with auto-fluorescent tissues.

Addressing these limitations through large collaborative networks and shared methodological standards will be essential to advance human brain research and its translational applications.

## 7 Conclusion

Human organotypic brain slice cultures (OBSC) present substantial challenges in terms of accessibility, reproducibility, and translational compatibility with existing tools. However, giant steps have significantly improved culture conditions, extending tissue viability, enabling systematic screening of viral serotypes and regulatory elements to target a broad range of cell types, allowing functional manipulation through optogenetics, and supporting the modeling of neurological pathologies such as Alzheimer's disease, Parkinson's disease, and brain tumors. This review highlights current and emerging approaches that could further enhance the potential of human OBSC, including gene editing strategies, chemogenetics, next-generation calcium and voltage indicators, and molecular biosensors. Finally, given that human OBSC have been parsimoniously utilized, there is a clear need for coordinated efforts to consolidate knowledge, share resources, and establish best practices within the field.
